# The Effects of COVID-19 on Healthcare Workers and Non-Healthcare Workers in Mexico: 14 Months into the Pandemic

**DOI:** 10.3390/medicina57121353

**Published:** 2021-12-10

**Authors:** César Esquivel-Chirino, Yolanda Valero-Princet, Luis Alberto Gaitán-Cepeda, Carlos Hernández-Hernández, Alejandro Macías Hernández, Hugo Laparra-Escareño, José Luis Ventura-Gallegos, Delina Montes-Sánchez, Ambar Lopéz-Macay, Fernando Hernández-Sánchez, William Alves de Oliveira, José Antonio Morales-González, Daniela Carmona-Ruiz, Karol Rosen-Esquivel, Alejandro Zentella-Dehesa

**Affiliations:** 1 Área de Básicas Médicas, División de Estudios Profesionales, Facultad de Odontología, Universidad Nacional Autónoma de México, Ciudad de México 04510, Mexico; 2 División de Ciencias de la Salud, Facultad de Odontología, Universidad Intercontinental, Ciudad de México 14420, Mexico; mvalero@uic.edu.mx; 3 Departamento de Medicina y Patología Oral Clínica, División de Estudios de Posgrado e Investigación, Facultad de Odontología, Universidad Nacional Autónoma de México, Ciudad de México 04510, Mexico; lgaitan@unam.mx; 4 Servicio de Estomatología, Instituto de Ciencias Médicas y Nutrición Salvador Zubirán, Ciudad de México 14080, Mexico; carlos.hernandezh@incmnsz.mx; 5 Área de Microbiología y Enfermedades Infecciosas, Departamento de Medicina y Nutrición, Facultad de Medicina, Universidad de Guanajuato, León 37670, Mexico; aaeemmhh@yahoo.com; 6 Departamento de Cirugía, Sección de Cirugía Vascular y Terapia, Instituto de Ciencias Médicas y Nutrición Salvador Zubirán, Ciudad de México 14080, Mexico; hugo.laparraesc@gmail.com; 7 Departamento de Medicina Genómica y Toxicología Ambiental, Instituto de Investigaciones Biomédicas, UNAM, Ciudad de México 04510, Mexico; venturag@iibiomedicas.unam.mx (J.L.V.-G.); azentell@iibiomedicas.unam.mx (A.Z.-D.); 8 Investigación Biomédica Básica, Licenciatura en Estomatología, Benemérita Universidad Autónoma de Puebla, Puebla 75770, Mexico; delina.montes@correo.buap.mx; 9 Laboratorio de Enfermedades Neuromusculares, 2do Piso de la Torre de Investigación, Instituto Nacional de Rehabilitación, Ciudad de México 14389, Mexico; lopez_macay@hotmail.com; 10 Departamento de Virología y Micología, Instituto Nacional de Enfermedades Respiratorias “Ismael Cosío Villegas”, Ciudad de México 04502, Mexico; fhernndezsnchez@gmail.com; 11 Investigación de la División de Ciencias de la Salud, Universidad Intercontinental, Ciudad de México 14420, Mexico; william.oliveira@uic.edu.mx; 12 División de Ciencias de la Salud, Facultad de Psicología, Universidad Intercontinental, Ciudad de México 14420, Mexico; 13 Laboratorio de Medicina de Conservación, Escuela Superior de Medicina, Instituto Politécnico Nacional, Ciudad de México 11340, Mexico; jmorales101@yahoo.com.mx; 14 Área de Ortodoncia, División de Estudios Profesionales, Facultad de Odontología, Universidad Nacional Autónoma de México, Ciudad de México 04510, Mexico; danyelacarmona@gmail.com; 15 Instituto de Ingeniería, Universidad Nacional Autónoma de México, Ciudad de México 04510, Mexico; karol_rosen@hotmail.com; 16 Unidad de Bioquímica, Instituto de Ciencias Médicas y Nutrición Salvador Zubirán, Ciudad de México 14080, Mexico

**Keywords:** COVID-19, cases, deaths, healthcare workers, physician, nurses, dentists, comorbidity

## Abstract

*Background and Objectives:* Healthcare workers (HCWs) play important roles in mitigating the COVID-19 pandemic and are more likely to become infected with COVID-19. Mexico, among other countries, had a high incidence and prevalence of cases and deaths from this disease. *Material and Methods:* This retrospective study evaluated the clinical characteristics as well as the geographical distribution of cases, deaths, and active cases of COVID-19 in HCWs and non-HCWs using official information from the Ministry of Health of Mexico. *Results:* A total of 235,343 cases of COVID-19 were reported in healthcare workers, and 2,094,191 cases were reported in non-healthcare workers. A total of 76.0% of cases in healthcare workers occurred in those who were between 25 and 50 years of age, and 71.4% of deaths occurred in those who were 50 to 69 years of age. Among healthcare workers, the most frequent comorbidities were obesity (15.2%), hypertension (10.9%), and diabetes (6.8%). Nurses were the group with the most cases (39.7%), followed by other healthcare workers (30.6%), physicians (26%), and dentists (1.6%). Physicians were the group with the most deaths (46%), followed by other professionals (30%), nurses (19%), and dentists (3%). *Conclusion:* These findings are likely the result of healthcare workers in Mexico being at a greater risk of exposure to SARS-CoV-2.

## 1. Introduction

In December 2019, a cluster of pneumonia cases of unknown etiology was identified in Wuhan, Hubei Province, China, a disease that was later named “COVID-19” [1]. The causal agent was identified and named “severe acute respiratory syndrome coronavirus” (SARS-CoV-2) [2]. Since then, the pandemic has been a substantial challenge for governments and health systems worldwide, including the for health system in Mexico [3].

The health system in Mexico has two segments: the first comprises The National Institutes of Health of Mexico, the Mexican Social Security Institute (IMSS, which is its acronym in Spanish), the Institute of Security and Social Services of Workers of the State (ISSSTE, which is its acronym in Spanish), the Secretariat of the Navy (SEMAR, which is its acronym in Spanish), the medical services of Mexican Petroleum (PEMEX, which is its acronym in Spanish), and the Secretariat of National Defense (SEDENA, which is its acronym in Spanish), and these services are for the portion of the Mexican population with social security coverage [4]. The second segment comprises the health services that are provided by the clinics, health centers, and hospitals of the Ministry of Health (SSA, which is its acronym in Spanish) and the Health Institute for Welfare (INSABI), which are intended for the portion of the Mexican population who does not have social security coverage. It was this segment that was responsible for Mexico’s public health system response to the COVID-19 pandemic [5].

During the evolution of the pandemic in Mexico, healthcare workers acquired the knowledge and skills that were necessary to manage patients with COVID-19 using the resources that were available. These professionals have faced numerous challenges since the beginning of the pandemic, including the conversion of hospitals for the management of patients who have been infected with SARS-CoV-2, insufficient personal protective equipment (PPE), and inadequate clinical protocols [6]. Additionally, within the health system, there were deficits of approximately 200,000 doctors and 300,000 trained nurses for the management and treatment of respiratory diseases [7,8].

Defined as any person who works in a health system, who has been in contact with patients, and who performs tasks to promote and protect health in their respective communities, healthcare workers (HCWs) have played important roles in combatting the COVID-19 pandemic [9]. This population group presented a higher probability of being infected with SARS-CoV-2 due to contact with infected patients [10,11].

The Mexican government used free molecular diagnostic testing, such as reverse transcription polymerase chain reaction (RT-PCR), and antigen tests in the National Network of Public Health Laboratories that had been recognized by the Institute of Epidemiological Diagnosis and Reference (InDRE, acronym in Spanish) to identify symptomatic who were suspected of being infected with COVID-19 [12]. The private health services commercialized the RT-PCR and antigen tests that could be used to identify COVID-19 cases in the general population at risk at a price.

Six months after the last week that was analyzed in this study, the COVID-19 cases and deaths have continued to increase in healthcare workers and non-healthcare workers. Since 28 February 2020, when the first case of COVID-19 was detected, and until 26 April 2021, 2,329,534 accumulated cases and 215,113 deaths have been reported in Mexico [13]. With respect to healthcare workers, in the period from January 2020 to 14 April 2021, the Pan American Health Organization (PAHO) reported 1,773,169 cases and 8665 deaths on the American continent. The nations with the maximum number of confirmed cases in healthcare workers were the United States, with 462,905 (26.0% of the total); Brazil, with 753,089 (42.4%); and Mexico, with 229,458 (12.9%). In contrast, Mexico ranked first in deaths, with 3534 (40.83%), followed by the United States, with 1538 (17.7%), and Brazil, with 656 (7.57%) [14].

In Mexico, the presence of one or more comorbidities, mainly diabetes, hypertension obesity, and asthma, had been reported as a risk factor for complications and death due to COVID-19 in HCWs and non-HCWs [15,16]. The objective of this study was to analyze the effects of COVID-19 on HCWs and non- HCWs in Mexico and to evaluate the clinical characteristics and geographical distribution of cases, active cases, and deaths during the period from 1 January 2020 to 26 April 2021.

## 2. Materials and Methods

### 2.1. Data Collection

This study had a retrospective design. Information on cases and deaths confirmed by RT-PCR tests and the clinical epidemiological association of COVID-19 cases and deaths for the study period from1 January 2020 to 26 April 2021 were analyzed. For this study, data for healthcare workers and for the general population (“non-healthcare workers”) were included. All data were obtained from the six official websites of the SSA as well as from the 475 Viral Respiratory Disease Monitoring Units that are distributed throughout the 32 states of the Mexican Republic. The data were integrated into the Epidemiological Surveillance System of Viral Respiratory Disease (SISVER, which is its acronym in Spanish), which is part of the National Epidemiological Surveillance System (SINAVE, which is its acronym in Spanish) of the Epidemiology Directorate of the SSA. A total of 130 daily technical communications were analyzed, detailing 54 epidemiological reports regarding the COVID-19 situation; 37 reports on healthcare workers with COVID-19; a SINAVE COVID-19 monitoring board; and a monitoring board for excess mortality due to COVID-19 belonging to the National Institute of Public Health of Mexico [17,18,19,20,21,22].

### 2.2. Operational Definition of the Variables

The operational definitions of cases and deaths from COVID-19 were used following the Standardized Guidelines for Epidemiological Surveillance as well as the guidelines of the Laboratory of Viral Respiratory Disease of the SSA [23], which were as follows:

- Infection disease—an illness caused by the transmission of a specific infectious biological agent from an infected person. The transmission can occur directly or indirectly from an infectious patient to a susceptible host [24].
- Suspected case of viral respiratory disease—an individual of any age who has with a presented cough, dyspnea, and fever accompanied by one or more of the following or symptoms: myalgia, arthralgia, odynophagia, chills, chest pain, rhinorrhea, polypnea, anosmia, dysgeusia, or conjunctivitis in the last 10 days.
- Laboratory-confirmed case of COVID-19—all patients that meet the definition of a suspected case of viral respiratory disease and who have received a diagnosis confirmed by an RT-PCR test and/or rapid antigen test performed in any of the National Network of Public Health Laboratories recognized by the Institute of Epidemiological Diagnosis and Reference (In-DRE, acronym in Spanish) of the SSA.
- Case of COVID-19 confirmed by epidemiological association—all patients that meet the definition of a suspected case and who have been shown to have been in close contact (proximity of less than one meter for 15 min or more, continuous or accumulated) with a person with a case confirmed by an RT-PCR test and/or rapid antigen test 2 to 14 days before the onset of symptoms, with the associated confirmed case registered on the SISVER platform.
- Active case of COVID-19—positive case with symptoms in the last 14 days.
- Death with laboratory-confirmed diagnosis—deceased person who meets the definition of a suspected case of viral respiratory disease confirmed by a laboratory RT-PCR test or by a rapid test (detection of SARS-CoV-2 antigen).
- Death due to COVID-19 confirmed by epidemiological association–deceased person who meets the definition of a suspected case of viral respiratory disease with a negative, nonamplified, or inadequate test or non-available sample, and who had contact with a laboratory-confirmed case (RT-PCR test or rapid antigen test) within 14 days before the date of symptom onset with the associated confirmed case registered on the SISVER platform.
- Healthcare workers—The information for HCWs was obtained by filtering data by “occupation” on the SISVER platform; the data on the platform were gathered from epidemiological reports regarding the COVID-19 situation in Mexico and in the reports on healthcare workers with COVID-19 by the SSA.
- “Occupation” was reported by respondents based on which group of healthcare workers that they belonged to, i.e., “physician”, “nurse”, “laboratory specialist”, “dentist”, and “other healthcare worker”.
- Comorbidities—The following comorbidities that are associated with COVID-19 were included: obesity, systemic arterial hypertension, diabetes mellitus, smoking, asthma, chronic obstructive pulmonary disease, asthma, immunosuppression, chronic renal failure, cardiovascular disease, and human immunodeficiency virus (HIV).
- Mortality rate—A measure of the frequency of the occurrence of death in a defined population during a specified interval of time [24].
- Excess mortality—the number of observed deaths minus the number of expected deaths and deaths associated with COVID-19 due to excess mortality—obtained by filtering data by “cause of death” on the National Registry of Population and Identity of the Mexican Republic [25].

### 2.3. Statistical Analysis

The variables sex, age group, comorbidities, and occupation are presented as per-centages for both groups: HCWs and non-HCWs. The infection and death rates were calculated by dividing 1 by the number of new infections per week and the number of new deaths.

For the statistical analysis, the nonparametric Wilcoxon test was selected for related samples, and the homogeneity of the groups was evaluated (null hypothesis). The data from two periods were compared: from May to November 2020 and from November 2020 to April 2021. Official data were only available for those months (May 2020 to April 2021). Because of that as well as to balance information, we formed two groups for this analysis. In all of the tables, values of *p* < 0.05 are considered to be statistically significant. All statistical analyses were performed using the program R (version 4.1.0 Camp Pontanezen).

## 3. Results

### 3.1. Distribution of COVID-19 Cases in Healthcare Workers and Non-Healthcare Workers in Mexico

Since 28 February 2020, when the first case of COVID-19 was detected, until 26 April 2021, 2,329,534 cases had been reported in Mexico: 235,343 had been reported in HCWs, and 2,094,191 had been reported in non-healthcare workers. Based on the seventeen epidemiological special reports regarding the COVID-19 situation that were produced by the SSA published on 26 April 2021, a total of 44.4% of the cases in healthcare workers were concentrated in five states; however, specific data were only available for Mexico City (14.5%, *n* = 34,314) and the State of Mexico (12.3%, *n* = 29,900). A total of 55.56% of the cases were distributed throughout the remaining 27 states. For healthcare workers, 27 states did not publish numerical data. Among non-healthcare workers, 53.0% of the cases were concentrated in five states: Mexico City (28.7%, *n* = 602,030), the State of Mexico (10.3%, *n* = 216,081), Guanajuato (5.5%, *n* = 115,822), Nuevo León (5.1%, *n* = 107,653), and Jalisco (3.3%, *n* = 70,542). A total of 46.9% (*n* = 982,157) of the cases were distributed throughout the remaining 27 states.

### 3.2. Active Cases of COVID-19 Cases in Healthcare Workers and Non-Healthcare Workers in Mexico

**Figure 1:**
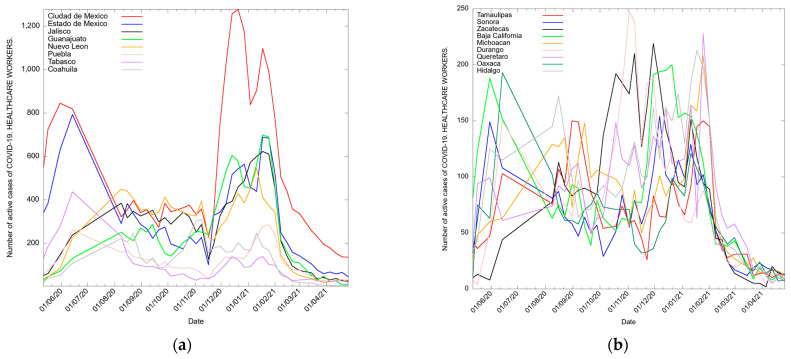

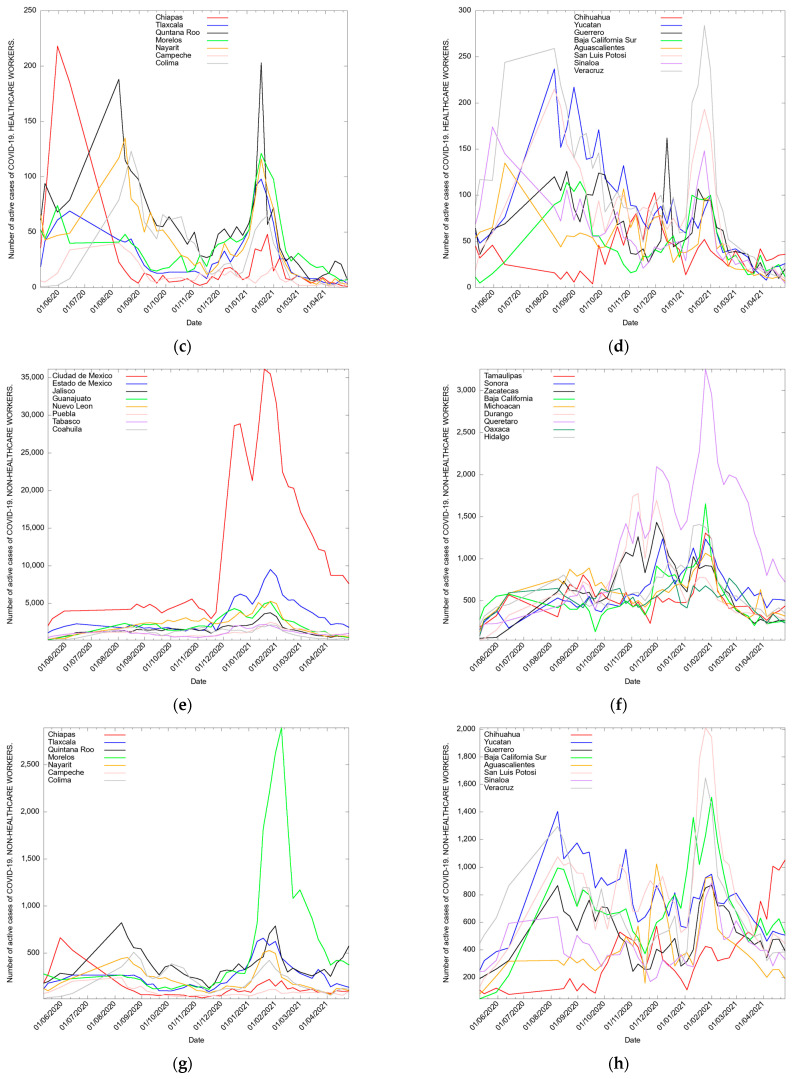
(**a**–**h**) shows the distribution of active cases among both groups in the 32 states of the Mexican Republic Active COVID-19 cases [26] healthcare workers (HCW) and (**e**–**h**) non-healthcare workers in Mexico confirmed between 12 May 2020 and 26 April 2021.

From 13 April to the 26, 2021, 21,525 active cases of COVID-19 were reported. Figure 1a–h shows the distribution of active cases among both groups in the 32 states of the Mexican Republic. Figure 1 shows the states with the highest number of active cases among HCWs. A total of 47.8% of the active cases were concentrated in five states: Mexico City (23.6%, *n* = 136), the State of Mexico (8.0%, *n* = 46), Chihuahua (6.2%, *n* = 36), Puebla (5.3%, *n* = 31), and Yucatan (4.5%, *n* = 26). A total of 52.1% of the active cases were distributed throughout the remaining 27 states. The states with the lowest number of active cases were Coahuila (0.5%, *n* = 3) and Chiapas (0.1%, *n* = 1). A decrease in the active cases in HCWs was observed at the beginning of February 2021, except for in the state of Chihuahua, where an increase in infections was identified.

Among non-HCWs, 20,950 active cases were reported. Figure 1e–h shows that 42.8% of active cases were concentrated in five states: Mexico City (35.7%, *n* = 7494), the State of Mexico (8.4%, *n* = 1776), Chihuahua (4.8%, *n* = 1016), Tabasco (4.7%, *n* = 985), and Quintana Roo (3.3%, *n* = 703). A total of 57.1% of active cases were distributed throughout the remaining 27 states. The states with the lowest number of cases were Chiapas (0.4%, *n* = 94) and Campeche (0.4%, *n* = 87). This was also the case for HCWs, where a decrease in active cases was also observed, except for in Chihuahua and Quintana Roo, where increasing trends were identified.

### 3.3. Epidemic Curve of COVID-19 Cases in Healthcare Workers and Non-Healthcare Workers in Mexico

**Figure 2:**
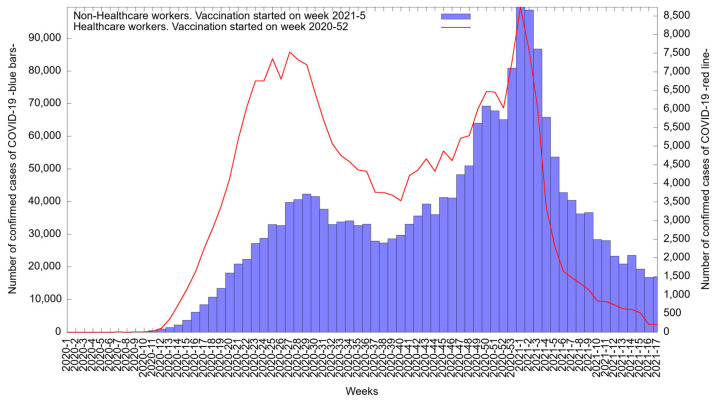
Epidemic curve of confirmed and suspected COVID-19 cases among healthcare workers (red, right-side axis) and non-healthcare workers (blue, left-side axis) between 1 January 2020 and 26 April 2021.

Figure 2 shows the epidemic curve of the COVID-19 cases reported in Mexico from 1 January 2020 to 26 April 2020 among both groups. At the beginning of the pandemic, there were few cases in HCWs, but cases increased, and a first peak was reached in week 27 of 2020 (in July), i.e., 7531 cases (3.2%).

Then, a consistent decrease was observed between weeks 29 and 40; however, a steady increase was again identified until the second peak was reached in week 1 of 2021 (in January), i.e., 8735 cases (3.7%). From week 2 to week 17 of 2021 (from January to April), a decrease in the number of cases in HCWs was observed.

At the beginning of the pandemic, there were less than a hundred cases per week among non-HCWs (blue); cases increased by week 12, with the first peak eventually being reached in week 29 of 2020 (in July), i.e., 42,269 cases (2.0%). Then, a consistent decrease in cases was observed until week 38 of 2020 followed by an increase until reaching a second peak in week 1 of 2021 (in January), i.e., 99,529 cases (4.7%). A decrease in cases was observed from week 2 to week 17 of 2021 (from January to April).

### 3.4. COVID-19 Infection Rate in Healthcare Workers and Non-Healthcare Workers in Mexico

**Figure 3:**
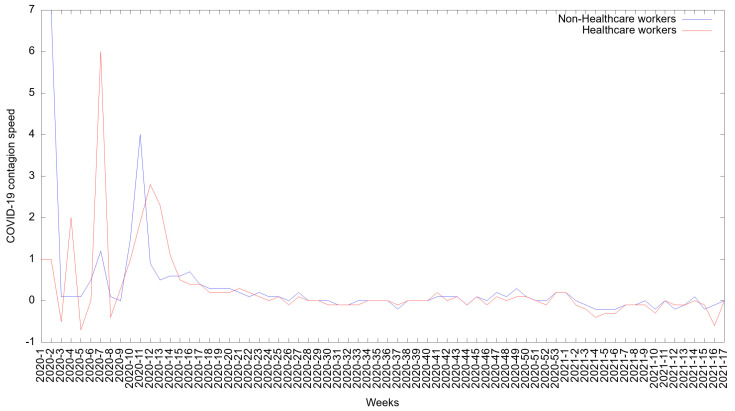
The speed of contagion of COVID-19 cases among healthcare workers and non-healthcare workers in Mexico between 1 January 2020 and 26 April 2021.

Figure 3 shows the COVID-19 infection rate among both groups. From week 9 of 2020 (in February), the infection rate for HCWs showed a constant increase. After week 12 (in March), the infection rate decreased and remained constant until week 15 of 2021 (in April). Thereafter, the infection rate decreased slightly.

Among non-HCWs, the infection rate was higher at the beginning of the pandemic, increasing rapidly between weeks 9 and 10 of 2020 (in February and March).

It decreased for two weeks until stabilizing in week 12 of 2020 (in March). Beginning in week 13 of 2020, the infection rate decreased slightly.

### 3.5. Deaths from COVID-19 in Healthcare Workers and Non-Healthcare Workers in Mexico

On 18 March 2020, the first death due to COVID-19 was confirmed in Mexico. As of 26 April 2021, 3829 deaths had been reported among HCWs. A total of 64.9% of the deaths (*n* = 2488) were concentrated in 10 states: Mexico City (20.9%, *n* = 803), the State of Mexico (9.2%, *n* = 354), Puebla (6.8%, *n* = 264), Veracruz (5.9%, *n* = 227), Jalisco (5.4%, *n* = 209), Guanajuato (4.3%, *n* = 166), Chihuahua (3.3%, *n* = 124), Hidalgo (3.0%, *n* = 115), Tabasco (2.9%, *n* = 114), and Sonora (2.9%, *n* = 112); about 35.0% of the deaths were distributed throughout the 22 remaining states. Similar to the reports detailing the number of cases, 22 states did not publish numerical data for COVID-19-related HCW deaths.

Among non-HCWs, 211,284 deaths were reported. A total of 46.4% were concentrated in five states: Mexico City (19.3%, *n* = 40,779), the State of Mexico (11.6%, *n* = 24,604), Puebla (5.2%, *n* = 11,006), Jalisco (5.4%, *n* = 11,512), and Guanajuato (4.8%, *n* = 10,308); about 53.5% of the deaths were distributed throughout the remaining 27 states. The states with the lowest number of deaths were Colima (0.5%, *n* = 1249) and Campeche (0.5%, *n* = 1136).

### 3.6. Epidemic Curve for Deaths Due to COVID-19 in Healthcare Workers and Non-Healthcare Workers in Mexico

**Figure 4:**
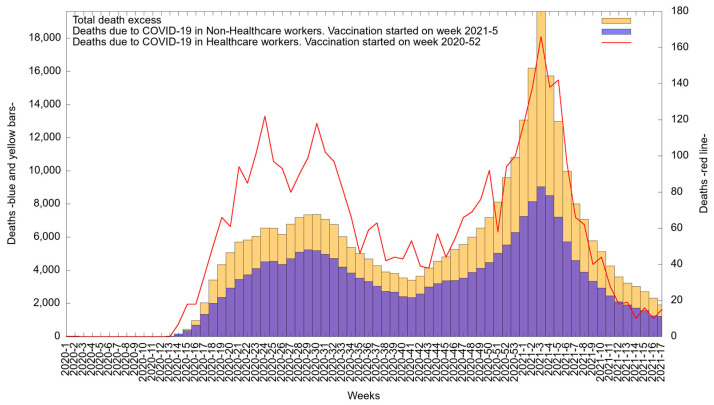
Epidemic curve of confirmed COVID-19 deaths/week among healthcare workers (red, right-side axis) and non-healthcare workers (blue) between 1 January 2020 and 26 April 2021 and total death excess for the same time period (yellow). Left-side axis for blue and yellow bars.

Figure 4 shows the epidemic curve for confirmed deaths among HCWs from 1 January 2020 to 26 April 2021. At the beginning of the pandemic, fewer than 10 deaths per week were observed in HCWs until week 14; from that point on, deaths increased until reaching a first peak in week 24 of 2020 (in June), i.e., 122 deaths/week (3.1%). Deaths decreased between weeks 25 and 27 of 2020 (in June) but reached a second peak in week 30 of 2020 (in July), i.e., 119 deaths/week (3.1%). Again, deaths decreased between weeks 31 and 35 of 2020 (in July and August). Between weeks 37 and 50 of 2020 (from September to December), there were four increases. Then, from week 52 of 2020 (in December) to week 2 of 2021 (in January), a continuous increase in deaths was observed, until a third peak was reached by week 3 of 2021 (in January), i.e., 166 deaths/week (4.3%). From week 4 to week 17 of 2021 (from January to April), a constant decrease was observed.

Among non-HCWs (blue), fewer than 10 deaths per week were observed until week 14; deaths increased until reaching a first peak in week 29 of 2020 (in July), i.e., 5234 deaths per week (2.4%). An increase was again identified, reaching a second peak in week 3 of 2021 (in January), with 9046 deaths per week (4.2%). A constant decrease was observed from weeks 4 to 17 of 2021 (from January to April).

A total of 346,855 deaths classified as excess mortality (yellow) were reported during the analyzed period. At the start of the COVID-19 pandemic in Mexico, there were less than 10 deaths per week; in week 14, deaths increased until a first peak was reached in week 30 of 2020 (in July), i.e., 7372 deaths (2.1%). An increase was again identified, reaching a second peak in week 3 of 2021 (in January), with 19,619 deaths per week (5.6%). A consistent decrease in deaths was observed from week 4 until week 17 of 2021 (from January to April).

### 3.7. The COVID-19 Death Rate in Healthcare Workers and Non-Healthcare Workers in Mexico

**Figure 5:**
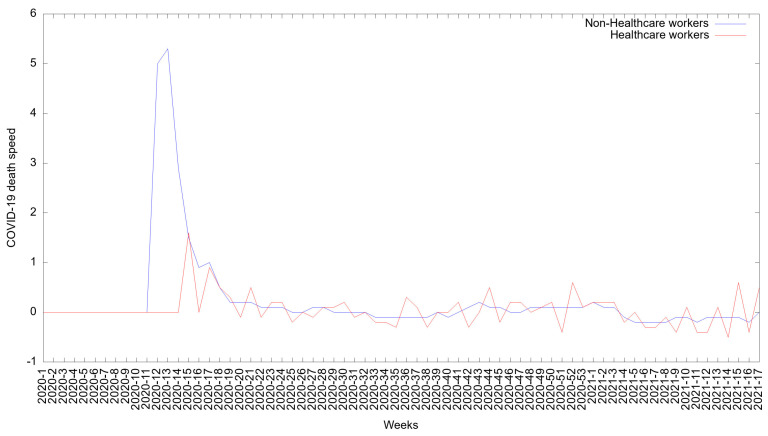
The speed of COVID-19 deaths among healthcare workers and non-healthcare workers in Mexico between 1 January 2020 and 26 April 2021.

Figure 5 shows the death rate for HCWs. At the beginning of the pandemic, the death rate was stable for both groups. The death rate for HCWs increased between week 16 of 2020 (in April) and week 17 of 2021 (in April). Following week 20 of 2020, the rate was relatively constant.

The death rate among non-HCWs rapidly increased from weeks 11 to 14 of 2020 (in March). It then decreased and remained stable from weeks 15 of 2020 to 17 of 2021 (in April).

Starting December 2020 (week 52 in in Figure 2 and Figure 4) universal vaccination for all frontline healthcare workers was provided free of charges from the Mexican government; this vaccination program only included Pfizer vaccines. A vaccination boost was applied within this period, and this vaccination process was officially concluded by April 2021.

### 3.8. Cases and Deaths Due to COVID-19 by Age Group in Healthcare Workers and Non-Healthcare Workers in Mexico

As seen in Table 1, 62.0% of the cases occurred in women, and the age group with the highest number of cases was 30 to 34 years of age (17.8%). Cases among non-HCWs were distributed equally between men and women (50.0%). The age group with the highest number of cases was 30 to 34 years of age (10.8%). A total of 69.0% of deaths among HCWs occurred in men, and the most affected age group was 60 to 64 years (16.9%). A total of 62.5% of deaths in non-HCWs occurred in men, and the most affected age group was 65 to 69 years of age (14.1%).

### 3.9. Cases and Deaths Due to COVID-19 and Comorbidities among Healthcare Workers and Non-Healthcare Workers in Mexico

New cases, deaths, and comorbidities among HCWs were analyzed by sex. The data were grouped according to the weekly COVID-19 reports. The first period was from May to November 2020, and the second report was from November 2020 to April 2021. Among non-HCWs, the highest number of cases was observed during the first period; however, the opposite was observed for the general population (see Table 2). Among HCWs, a significant decrease was observed in the number of COVID-19 cases in men during the second period. There were no other significant differences.

### 3.10. Comorbidities Related with COVID-19 Cases in Healthcare Workers and Non-Healthcare Workers in Mexico

The data on comorbidities related to COVID-19 cases were classified based on the number of comorbidities presented, i.e., one or more than one. As seen in Table 3, obesity (15.2%), hypertension (10.9%), and diabetes (6.8%) were the most prevalent comorbidities among HCWs. Similarly, hypertension (17.2%), obesity (14.3%), and diabetes (13.3%) were the most prevalent comorbidities among non-HCWs.

### 3.11. Cases and Deaths Due to COVID-19 by Occupation in Healthcare Workers and Non-Healthcare Workers in Mexico

As seen in Table 4, the health occupation with the highest number of cases was nursing, i.e., 93,432 cases (39.7%), followed by other professionals, i.e., 72,015 cases (30.6%), physicians, i.e., 61,189 cases (26.0%), and dentists, i.e., 3765 cases (1.6%). The most deaths occurred among physicians, i.e., 1761 deaths (46.0%), followed by other professionals, i.e., 1149 deaths (30.0%), and female nurses, i.e., 728 deaths (19.0%). The results shown in Table 4 complete the epidemiological panorama that was included in the 23rd Epidemiological Report of the COVID-19 Situation produced by the SSA regarding the professions with the most cases of COVID-19; until then, there were no data reported for weeks 23 to 53 of 2020 and weeks 1 to 9 of 2021.

### 3.12. New Cases of and Deaths Due to COVID-19 by Period among Healthcare Workers in Mexico

No significant differences were observed in the periods analyzed for HCWs and non-HCWs (Table 5).

### 3.13. Health System Personnel Combatting the Pandemic in Mexico

Table 6 shows the healthcare workers combatting the pandemic as reported by the Subsystem of Information of Equipment, Human Resources and Infrastructure for Health Care (SINERHIAS, which is its acronym in Spanish). From March to December 2020, the number of health personnel increased from 151,743 to 178,176. This increase was due to the hiring of healthcare workers by the Institute of Health and Welfare of the SSA, SEMAR, ISSSTE and IMSS exclusively to combat COVID-19. By mid-2021, some of these healthcare workers completed their temporary contracts. Additionally, the IMSS conducted a special program known as “Operation Chapultepec”, which allowed the recruitment of another 620 healthcare workers to support clinical care in this institution; however, none of these contracted healthcare workers were subspecialists.

## 4. Discussion

The main objective of this study was to describe the effects of COVID-19 on healthcare workers in Mexico in terms of the confirmed cases and deaths in a 14-month period since the onset of the pandemic. Our data showed no differences regarding new cases and deaths, the transmission rate, the epidemic curve, and the death rate between HCWs and non-healthcare workers. In the case of the geographical distribution of cases and deaths, most of them were concentrated in five states, i.e., Mexico City, the State of Mexico, Jalisco, Guanajuato, and Nuevo León. Hospitals converted for COVID-19 care were also predominant in these states.

The SSA reported that HCWs were the profession with the highest number of infections at the beginning of the pandemic [26,27,28,29]. Our data support this fact. During the first wave of the pandemic and at its onset, Mexican HCWs had [16,17,18,19] more infections than non-HCWs, while in the second wave, there was a decrease in active cases among HCWs. Establishing the causes of these changes beyond the principal objective of the present report could be reflected as a learning curve. It is undoubted that the implementation of biosecurity protocols, prevention strategies, and methods for early diagnosis improved over the course of the pandemic.

The COVID-19 transmission rates were similar for HCWs and non-HCWs. These data could be explained from several points of view. It is now known that SARS-CoV-2 has higher infectivity than other viruses and pathogens such SARS-CoV-2, Middle Eastern respiratory syndrome coronavirus (MERS-CoV), and H1N1 influenza [30]; however, at the beginning of the pandemic, the routes of transmission of SARS-CoV-2 had not been fully assessed. As a consequence, the virus could be transferred from home to the workplace and vice versa or within families and/or social nuclei. On the other hand, similar transmission rates could reflect inadequate biosafety protocols, specifically in the pre-pandemic era; however, in Mexico, infectious diseases such as tuberculosis and dengue are highly prevalent; therefore [31,32], biosecurity protocols for attending to infectious patients should be established.

The occurrence of deaths due to COVID-19 was similar in both groups. In the first and second waves of the pandemic, three peaks were identified: the first was identified in week 24 of 2020; the second was identified in week 30 of 2020; and the third was identified in week 3 of 2021. The third peak corresponded to the greatest increase in HCWs deaths due to COVID-19. This result is striking because at that time, there were well-developed biosafety protocols for the management of COVID-19 patients as well as better knowledge about the disease and its viral biology. Although we are unable to determine the causes of the increase in delinquency to date, it is likely to have been the result of the sum of several factors, including comorbidities.

At the moment of writing this report, the deaths directly related to COVID-19 among HCWs represented 1.7% of the total number of COVID-19- related deaths. This figure is even more significant because it means that 2.1% of the total number of HCWs (178,176 HCWs) in Mexico assigned to attending the COVID-19 pandemic had died [33] (Table 5).

The results are consistent with the data reported by the PAHO; Mexico ranked first in HCWs, followed by the United States, Peru, Brazil, Argentina, and Bolivia [34]. The results obtained indicate that working in the health sector during the COVID-19 pandemic in Mexico constituted a greater risk of infection and death. These data are consistent with those reported by Van der Plaat et al. [35], who found that HCWs who worked with infected patients had a higher risk of infection than HCWs who did not have direct contact with COVID-19 patients did.

After analyzing cases of and deaths due to COVID-19 based on age and sex, demographic differences were identified between both groups. Among the non-health-related population, 62% of the cases involved women; among HCWs, the distribution by sex was equitable (50%). Similarly, the distribution of cases by age group was different: among HCWs, 76.0% of cases involved individuals between 25 and 50 years; in contrast, 52.2% of cases among non-HCWs involved individuals in the same age group. This age difference is probably due to the age of the HCWs; most of these personnel are between 25 and 50 years of age, with a decrease after 50 years due to public health policies. Notably, 12% of cases in non-HCWs occurred in people under 25 years of age.

The distribution of deaths among HCWs by age affected health sector operations. A total of 71.4% of deaths due to COVID-19 among HCWs occurred in individuals between 50 and 69 years of age. These results could have an impact on the health sector because of the decrease in experienced workers. Death among those who were 40 and 60 years of age accounted for 31.2% of all HCW deaths. Regardless of the numerical impact, the number of deaths presumably implies consequences, such as loss of professional experience and organizational leadership, both of which would have direct effects on the health system. This impact will last between five and ten years, the time required for new generations of HCWs to acquire experience and to build capacity. HCWs did not have a predisposition to infection that was related to sex, and the greater infection and death rates for younger age groups were not related to health. These facts are probably related to working age in addition to self-care more than risk factors or the presence of different comorbidities.

An important consideration in the analysis of deaths is excess mortality. As seen in Figure 4, excess mortality peaked at week 17 of 2021. Deaths due to COVID-19 represented 70.9% of deaths from all causes [25]. Unfortunately, although these data include HCWs, other labor sectors were not specifically identified; therefore, it was not possible to determine how much of this excess mortality was associated with the health care sector.

During the period that was studied, differences were observed in the number of new cases, deaths, and comorbidities between both groups. Among non-HCWs, new cases increased by 78.7% and in HCWs, and then they decreased by 54.6%. This decrease may be attributable to improvements in protocols and patient management; however, this information should be analyzed with attention because the periods are not equal: there were fewer days in the second period, leading to fewer cases. Indeed, a decrease in new cases was identified between November 2020 and April 2021; however, between November 2020 and February 2021, the largest numbers of cases and deaths were observed.

Comorbidities are associated with complications in patients who have been infected with SARS-CoV-2. Obesity, hypertension, diabetes, and acute respiratory failure are factors that directly influence patient prognosis. In this study, diabetes, hypertension, and obesity were the comorbidities in both groups. In Mexico, these comorbidities represent important risk factors that are associated with COVID-19 and that represent substantial relevance because there are 12.8 million diabetic individuals and approximately 30 million hypertensive individuals in Mexico [36,37]. In fact, diabetes is considered a disease of national public health importance. Likewise, Mexico has one of the highest rates of obesity among all countries [38]. Thus, the endemic presence of these comorbidities may explain the high mortality rates from COVID-19 that were observed in Mexico. Among both groups, those with COVID-19 who presented at least one comorbidity reported the same four comorbidities: obesity, hypertension, diabetes, and smoking. However, the distribution of these comorbidities was different between the two groups: obesity accounted for 45.6% of the comorbidities reported by HCWs and 32.4% of the comorbidities reported by non-healthcare workers. In contrast, there were more hypertensive and diabetic patients among non-HCWs than among the group of HCWs. Smoking accounted for 19.3% of the total comorbidities reported by HCWs, a figure higher than the one of 16.5% that was reported by the non-HCWs. However, none of these differences were statistically significant. These results were consistent with the study by Guerrero-Torres et al. [39], who reported that the presence of these comorbidities could be a risk factor for complications in HCWs who had COVID-19 in Mexico City. Our data showed that there were a greater number of cases among HCWs without comorbidities. This reduction in comorbidities in HCWs is probably associated with better health education associated with academic training or the hospital environment.

The SSA reported that HCWs were the professionals with the highest number confirmed of COVID-19 cases and deaths at the beginning of the COVID-19 pandemic. It is unknown whether the severity of the disease was greater among physicians or what factors predisposed physicians to death. It is also unknown whether protective factors played a role in the lower number of deaths among nurses.

For dentists, the difference in the distribution of cases and deaths, compared to those for other health professions, could be associated with confinement and a decrease in activities during the pandemic. Public and private dental care services were suspended or decreased, translating into dentists having a lower risk of exposure to SARS-CoV-2. However, these professionals are directly exposed to saliva and perform treatments that generate aerosols that can spread SARS-CoV-2; consequently, it is significant to conduct studies in this area of health care [40,41].

The vaccination of healthcare workers overlapped with the second peak of COVID-19 cases, and it was noteworthy that the rates of contagion and death showed descriptive decreases after week 53, but this change was present in both healthcare workers and non-healthcare workers (Figure 3 and Figure 5). The Mexican Ministry of Health approved the National Vaccination Plan for COVID-19, which was universal, free, and voluntary, in December 2020 [42]. After the beginning of the vaccination and until 26 April 2021, 6,501,739 million COVID-19 vaccine doses had been administered: 970,264 thousand doses of the Pfizer-BioNTech vaccine had been administered in frontline healthcare workers, 10,636,571 million doses of the Pfizer-BioNTech, Sinovac, and Sputnik vaccines had been administered in adults of 50 years or older; and 447,186 thousands doses of CanSino vaccine had been administered in educational personnel [43].

There is no detailed information to determine whether those who work in the in-formal sector, such as housewives, transport operators, food supply personnel in public markets, etc., had a higher risk of infection than HCWs did.

The present study has limitations. One of them is the nature of the databases. The data were obtained from official websites and technical communications. The information offered is the result of a compilation of information. Therefore, it was not possible to perform complex statistical analyses. Therefore, this study was only descriptive. It is necessary that official sources of information create complete databases that are available so that statistical analyses can be performed to provide associations.

## 5. Conclusions

The numbers of cases among HCWs account for 10.1% of all COVID-19 cases in Mexico. This finding demonstrates the magnitude of exposure and risk to which HCWs have been subjected in Mexico. This percentage is substantial, especially when considering that Mexico has a total of 126,014,024 inhabitants.

COVID-19 cases and deaths among HCWs than non-HCWs at the beginning of the COVID-19 pandemic represent the highest number of cases and deaths among any other profession. In fact, among the various professional sector, physicians and nurses continue to have the highest probability of contagion in the pandemic, a finding linked to the important role that they play during a health care crisis.

The statistical analysis performed on free access official data published by the Mexican Government is a straight-forward approach to address the main problems affecting HCWs in our country that allowed the cases and deaths associated with COVID-19 to be documented.

Currently, the issues associated with the initial strains of the virus seem to be dissipating, but issues with new strains are intensifying. It is likely that there will still be a higher number of cases among non-HCWs than HCWs. Therefore, it will be important to analyze the effect of vaccinations on cases and deaths due to COVID-19 that occur during a third wave.

## Figures and Tables

**Table 1:** COVID-19 cases and death numbers by gender and age in healthcare workers and non-healthcare workers in Mexico.

	Cases HCWs	%	Cases Non-HCWs	%	Deaths HCWs	%	Deaths Non-HCWs	%
Gender								
Female	145,913	62	1,017,792	50	1187	31	80,679	37.50
Male	89,430	38	1,076,399	50	2642	69	134,434	62.50
Age groups								
<25	9386	3.99	283,650	12.18	5	0.13	1455	0.68
25–29	36,569	15.54	244,530	10.50	39	1.02	1603	0.75
30–34	42,090	17.88	253,137	10.87	104	2.72	3077	1.43
35–39	39,531	16.80	244,772	10.51	164	4.28	5104	2.37
40–44	33,174	14.10	232,282	9.97	275	7.18	8522	3.96
45–49	27,492	11.68	241,293	10.36	416	10.86	14,224	6.61
50–54	20,683	8.79	213,915	9.18	492	12.85	19,204	8.93
55–59	13,474	5.73	181,431	7.79	613	16.01	25,206	11.72
60–64	7524	3.20	139,753	6.00	647	16.90	29,720	13.82
65–69	3432	1.46	105,802	4.54	566	14.78	30,443	14.15
70–74	1214	0.52	77,002	3.31	286	7.47	27,609	12.83
75–79	456	0.19	53,345	2.29	125	3.26	21,931	10.20
80–84	187	0.08	32,785	1.41	62	1.62	14,877	6.92
85–89	86	0.04	17,296	0.74	28	0.73	8327	3.87
90–94	30	0.01	6356	0.27	7	0.18	2975	1.38
>94	15	0.01	2185	0.09	0	0.00	836	0.39
TOTAL	235,343	100	2,094,191	100	3829	100	211,284	100

**Table 2:** COVID-19 cases and deaths in healthcare workers and non-healthcare workers in Mexico in the two analyzed periods.

	HCWs			Non-HCWs		
Variable	May 2020–1st Half November 2020 (Mean)	2nd Half November 2020–April 2021 (Mean)	*p*-Value	May 2020–1st Half November 2020 (Mean)	2nd Half November 2020–April 2021 (Mean)	*p*-Value
Total new cases COVID-19	7909	3857	0.105	40,507	72,426	0.103
Female new cases	4817	2479	0.189	19,891	36,512	0.076
Male new cases	3093	1379	* 0.040	20,616	35,911	0.168
New cases with at least 1 comorbidity	2689	1249	0.177	17,776	31,866	0.089
New cases with no comorbidity	5220	2608	0.294	22,731	40,557	0.119
Total new deaths	105	86	0.380	4549	5117	0.432
Female new deaths	32	28	0.218	1733	1825	0.892
Male new deaths	74	58	0.387	2816	3233	0.321

In this table, data size for non-healthcare workers, equals 42, 21 for each period for healthcare workers, data size equals 40,20, for each period. * *p*-values below 0.05 are considered statistically significant in a two-sided test.

**Table 3:** Present comorbidities among healthcare workers and non-healthcare workers associated with COVID-19 in Mexico in the analyzed period.

	Cases HCWs	%	Cases Non-HCWs	%
Comorbidities				
None	156,597	66.54	1,172,747	56
Cases with at least 1 or more	78,746	33.46	921,444	44
Obesity	35,960	15.28	299,469	14.3
Hypertension	25,817	10.97	360,201	17.2
Diabetes	16,097	6.84	278,527	13.3
Smoking	15,250	6.48	152,876	7.3
Asthma	8543	3.63	46,072	2.2
COPD	2524	1.08	31,413	1.5
Immunosuppression	1506	0.64	16,754	0.8
Chronic kidney disease	1153	0.49	31,413	1.5
Cardiovascular disease	871	0.37	23,036	1.1
HIV/AIDS	541	0.23	6238	0.3

**Table 4:** Numbers of COVID-19 cases and deaths by occupation type in healthcare workers in Mexico.

Occupation	Cases HCWs	%	Deaths HCWs	%
Nurses	93,432	39.7	728	19
Physicians	61,189	26	1761	46
Laboratory personnel	4942	2.1	77	2
Dentist	3765	1.6	114	3
Others	72,015	30.6	1149	30
Total	235,343	100	3829	100

**Table 5:** New COVID-19 cases and deaths by occupation in healthcare workers.

	Healthcare Workers by Occupation		
Variable	May 2020–1st Half November 2020 (Mean)	2nd Half November 2020–Apr 2021 (Mean)	*p*-Value
Physicians total new cases	2057	1003	0.097
Dentists total new cases	79	109	0.576
Nurses total new cases	3243	1429	0.067
Others total new cases	2373	1228	0.189
Physicians total new deaths	52	36	0.852
Dentists total new deaths	2	2	0.363
Nurses total new deaths	19	17	0.478
Others total new deaths	31	27	0.575

In this table for healthcare workers, data were sampled from 40 weekly reports, and 20 obtained for each analyzed period; the data size for non-healthcare workers equals 42—21 for each period. *p*-values below 0.05 are considered statistically significant in a two-sided test.

**Table 6:** Healthcare workers assigned to care for COVID-19 in Mexico.

Type Personnel	SINERHIAS IMSS, ISSSTE March 2020	INSABI * April 2020	SEMAR September 2020	ISSSTE September 2020	Operation Chapultepec-IMSS December 2020
Physicians	37,596	--	--	--	--
Nurses in contact	112,042	--	--	--	--
Urgenciologists	1284	--	--	--	--
Pulmonologists	207	--	--	--	--
Infectologists	174	--	--	--	--
Epidemiologists	440	--	--	--	--
Supportive healthcare workers	--	17,563	--	--	620
Hiring healthcare workers in hospitals	--	4039	500	4331	--
Total	151,743	21,602	500	4331	* 620

* Some workers associated with the IMSS were hired to support other entities; therefore, those workers were not considered additional healthcare workers, nor were the same HCWs working more hours. SINERHIAS: Human Resources and Infrastructure for Health Care; IMSS: Mexican Social Security Institute; ISSSTE: Institute of Security and Social Services of Workers of the State; SEMAR: Secretariat of the Navy; INSABI: Health Institute for Welfare

## Data Availability

No permission was necessary to obtain the data used in this study since they were made public and remained open access once published on the official website of the Ministry of Health of Mexico. The protocol research was conducted in full accordance with the World Medical Association Declaration of Helsinki.
